# AI in Medicine: Uncovering the Educational Pulse Through Students’ Perspective

**DOI:** 10.7759/cureus.109100

**Published:** 2026-05-18

**Authors:** Sarita K Sharma, Amit Mohan Mandhare, Pragati G Rathod, Neethu Baby, Shubham S Bhawane

**Affiliations:** 1 Community Medicine, Government Medical College, Nagpur, IND; 2 General Surgery, Prakash Institute of Medical Sciences & Research, Urun-Ishwarpur, IND

**Keywords:** ai literacy, artificial intelligence, curriculum development, digital health education, healthcare technology adoption, medical education, medical student attitudes, medical students, perceptions

## Abstract

Background

AI is rapidly transforming healthcare delivery and medical training. While global bodies advocate for incorporating AI competencies into medical curricula, limited data exist on the perceptions, knowledge levels, ethical concerns, and training needs of Indian medical students. Understanding student perspectives is vital for designing future-ready curricula.

Methodology

A descriptive cross-sectional study was conducted among MBBS students at Government Medical College, Nagpur, between August and September 2024. A pretested, semi-structured, self-administered Google Form questionnaire (Google LLC, Mountain View, CA, USA) assessed perceptions of AI utility, ethical concerns, self-rated knowledge, and preferences for AI training. A convenience sample was obtained by inviting ~50 students from each academic phase (response rate: 94.4%, n = 236/250). Data were analyzed using IBM SPSS Statistics for Windows, version 25.0 (released 2017; IBM Corp., Armonk, NY, USA). Ethical approval was obtained from the Institutional Ethics Committee, and informed consent was collected digitally.

Results

A total of 236 students participated (mean age 20.52 ± 1.47 years; 53% male). Most respondents expressed positive perceptions of AI’s potential to enhance decision-making, improve precision, and support healthcare accessibility (≥60% agreement). However, concerns about erosion of humanistic care, compromise of patient-physician relationships, and data privacy breaches were prominent. Only 31 students (13.1%) had received prior AI training. Students without training were significantly more likely to believe that AI threatens physician employment (71.2% vs. 35.5%; p = 0.00042), whereas trained students viewed AI as an augmentative tool and felt more confident about using AI in future practice (p < 0.00001). Self-rated knowledge was low, with 66.5% reporting no or minimal familiarity. A substantial majority (78.8%) expressed willingness to receive AI training. Students strongly supported integrating practical AI skills, ethics, research applications, predictive modeling, and clinical decision-support tools into the curriculum (≥60% endorsement across domains). Online learning was the preferred mode (38.1%).

Conclusions

Medical students demonstrate high enthusiasm for AI but possess limited knowledge and significant ethical concerns. Prior exposure to AI training correlates with more informed, positive perceptions of AI as a supportive rather than a replacement technology. The strong demand for a structured AI curriculum highlights the need for integrating foundational, technical, and ethical AI competencies into undergraduate medical education. A balanced, context-sensitive curriculum emphasizing both technological literacy and humanistic values is essential for preparing future physicians for AI-enabled healthcare.

## Introduction

The rapid advancement of AI technologies is profoundly reshaping the landscape of healthcare, influencing not only clinical practice but also the training of future physicians. AI applications, ranging from diagnostic algorithms to personalized treatment planning, have demonstrated significant potential to enhance patient outcomes, reduce medical errors, and improve healthcare accessibility [1,2]. Recognizing these transformative capabilities, leading organizations such as the World Medical Association and the Association of American Medical Colleges have advocated for the integration of AI education into medical curricula to ensure that graduates are equipped to navigate and leverage these emerging tools effectively [3,4].

In medical education, AI offers numerous benefits, including the creation of immersive, multisensory learning environments, provision of real-time feedback, facilitation of problem-based learning, and the opportunity to engage in risk-free simulations of complex medical scenarios [5]. However, the successful incorporation of AI into medical training hinges not only on technological adoption but also on the perceptions and readiness of medical students, the future healthcare workforce. Understanding students’ attitudes toward AI, their perceived benefits, and their concerns, particularly regarding ethical implications and the potential erosion of the humanistic aspects of medicine, is essential for designing effective educational strategies [6,7].

Despite the growing enthusiasm for AI, there remains a paucity of data on how medical students perceive its role, benefits, and challenges within the context of their education and future practice. Addressing this gap is critical, as students’ perspectives can inform curriculum development and help educators balance the promise of technological innovation with the preservation of core medical values.

Hence, the present study was carried out with the primary objective of assessing medical students’ perceptions of AI in medical education and healthcare, focusing on perceived benefits and risks, including ethical concerns and fear of human replacement, and with the secondary objectives of assessing attitudes toward AI training and curriculum integration and examining the association between prior AI training and perceptions of AI. Thus, by uncovering the educational pulse through student perspectives, this research seeks to guide the integration of AI into medical curricula in a manner that is both effective and ethically grounded.

## Materials and methods

Study design and setting

This was a descriptive cross-sectional questionnaire-based study conducted at Government Medical College, Nagpur, a premier tertiary care teaching institute in Central India. The study was carried out over a two-month period from August to September 2024 and was designed to assess undergraduate medical students’ awareness, perceptions, ethical concerns, and training needs related to AI in medical education.

Study population and sample size

Approximately 50 students from each academic phase (first professional, second professional, third professional part 1, third professional part 2, and interns) were invited using convenience sampling to ensure uniform representation across all phases of MBBS training. Recruitment was conducted through institutional WhatsApp groups (Meta Platforms, Menlo Park, California, USA), classroom announcements, and official student communication channels. Two reminder messages were circulated at one-week intervals during the study period to improve response rates. Only those who voluntarily completed the Google Form (Google LLC, Mountain View, CA, USA) were considered, and entries that were incomplete or inaccurate were excluded. A total of 250 students were approached, of whom 236 submitted complete responses (response rate: 94.4%). Fourteen incomplete or duplicate responses were excluded prior to analysis.

Eligibility criteria

All MBBS students enrolled at the institution who consented to participate and submitted completed questionnaires were included, while those with incomplete or duplicate responses and those who did not provide informed consent were excluded from the analysis.

Data collection instrument

Data were collected using a pretested, semi-structured, self-administered questionnaire developed in Google Forms (Appendix A). The instrument was constructed based on a review of validated tools from previously published studies and expert input from faculty in community medicine, medical education, and clinical informatics.

The questionnaire comprised five key sections: sociodemographic profile; perceptions regarding the utility of AI in medical education and practice; concerns related to AI ethics and data privacy; training needs and preferences for AI education; and self-assessment of previous knowledge about AI. Perceptions were assessed using a 5-point Likert scale, where 0 indicated “strongly disagree,” and 4 indicated “strongly agree.”

Instrument validation and pilot testing

Content validity was established by a panel of five experts in medical education and public health. A pilot study was conducted with 20 medical students to assess clarity, internal consistency, and face validity of the instrument. Cronbach’s alpha was calculated for internal consistency of the Likert items and found to be acceptable (>0.7). Data from the pilot were excluded from the final analysis.

Ethical considerations

Ethical clearance was obtained from the Institutional Ethics Committee, Government Medical College, Nagpur (3744/EC/Pharmac/GMC/NGP). Participation was entirely voluntary. Digital informed consent was obtained prior to questionnaire access. All responses were anonymized and stored securely, ensuring compliance with data privacy and ethical research standards.

Data management and analysis

Data were downloaded from Google Forms in Excel format (Microsoft Corporation, Redmond, WA, USA) and imported into IBM SPSS Statistics for Windows, version 25.0 (released 2017; IBM Corp., Armonk, NY, USA) for analysis. Descriptive statistics (frequencies, percentages, means, and standard deviations) were used to summarize student characteristics. Likert-scale responses were treated as ordinal variables and summarized using frequencies and percentages. Composite perception scores were assessed for approximate normality before applying parametric tests such as independent-sample t-tests or ANOVA. Chi-square tests were used for categorical variables. A p-value < 0.05 was considered statistically significant.

## Results

**Figure 1:**
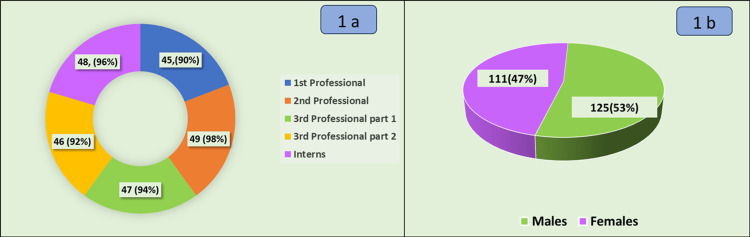
Distribution of study participants as per their academic year and gender

Figure 1 displays the distribution of study participants as per academic year of study in MBBS and gender. There was almost equal representation from all academic years. Of the 236 participants, 125 (53%) were males, and 111 (47%) were females, with a mean age of 20.52 (±1.47) years.

**Figure 2:**
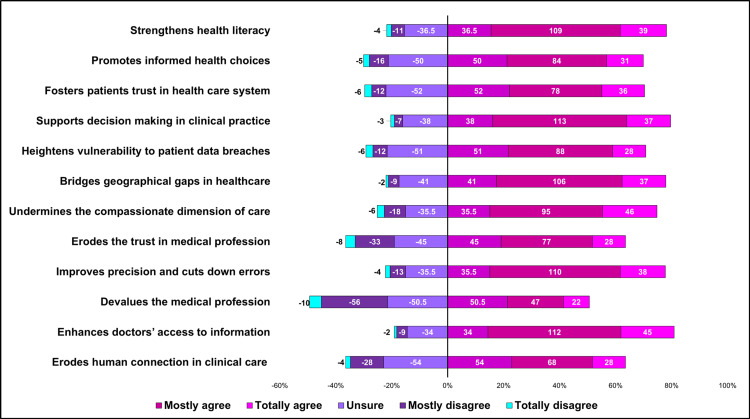
Frequency distribution of perceptions of AI in medicine

Figure 2 illustrates the frequency distribution of perceptions regarding the role of AI in medicine across a spectrum of positive and negative attributes. A majority of respondents expressed favorable views on AI’s potential to strengthen health literacy, promote informed health choices, foster patient trust in the healthcare system, support clinical decision-making, and improve precision while reducing errors, with more than 60% either totally or mostly agreeing with these statements.

Similarly, positive perceptions were noted regarding AI’s ability to bridge geographical gaps in healthcare and enhance doctors’ access to information. However, concerns were also evident, with over 50% of respondents agreeing that AI may heighten vulnerability to patient data breaches, erode human connection in clinical care, and undermine the compassionate dimension of care.

Notably, substantial disagreement was observed for statements suggesting that AI devalues the medical profession and erodes trust in the medical profession, indicating that most participants do not perceive AI as a threat to the professional stature of clinicians. A smaller yet significant proportion of respondents remained unsure across several domains, reflecting ongoing uncertainty or a need for further awareness about AI’s integration in clinical settings.

Out of 236 participants, 31 (13.1%) had received training in AI, while the remaining 205 (86.9%) had not received any training. Table 1 demonstrates the relationship between prior AI training and perceptions of its impact on medical practice. Respondents without AI training were significantly more likely to believe that AI reduces the need for physicians and threatens employment (71.2% vs. 35.5%; p = 0.00042). Participants with training were more likely to view AI as a supportive tool rather than a replacement (p = 0.00005) and felt more confident about becoming better doctors through AI applications (p < 0.00001). Additionally, they more often agreed that AI tools can improve efficiency in maintaining patient records (38.7% vs. 23%; p < 0.00001).

**Table 1:** Relationship between previous training in AI and selected statements of perceptions ^*^ Statistically significant (p < 0.05)

Statements		Training received in AI		p-Value
		Yes (n = 31) (%)	No (n = 205) (%)	
“The use of AI in medicine reduces the need for physicians and, thus, employment opportunities.”	Agree	11 (35.5)	146 (71.2)	0.00042^*^
	Disagree	9 (29)	25 ((12.1)	
	Unsure	11 (35.5)	34 (16.7)	
“It cannot replace the physician, but it can help him.”	Agree	17 (54.9)	158 (77)	0.00005^*^
	Disagree	5 (16.1)	3 (1.5)	
	Unsure	9 (29)	44 (21.5)	
“I think I will be a better doctor with the widespread use of AI applications.”	Agree	16 (51.6)	194 (94.6)	<0.00001^*^
	Disagree	4 (12.9)	10 (4.9)	
	Unsure	11 (35.5)	2 (0.5)	
“I feel AI-powered tools can be used to maintain patient records efficiently.”	Agree	12 (38.7)	47 (23)	<0.00001^*^
	Disagree	3 (9.7)	132 (64.3)	
	Unsure	16 (51.6)	26 (12.7)	

**Figure 3:**
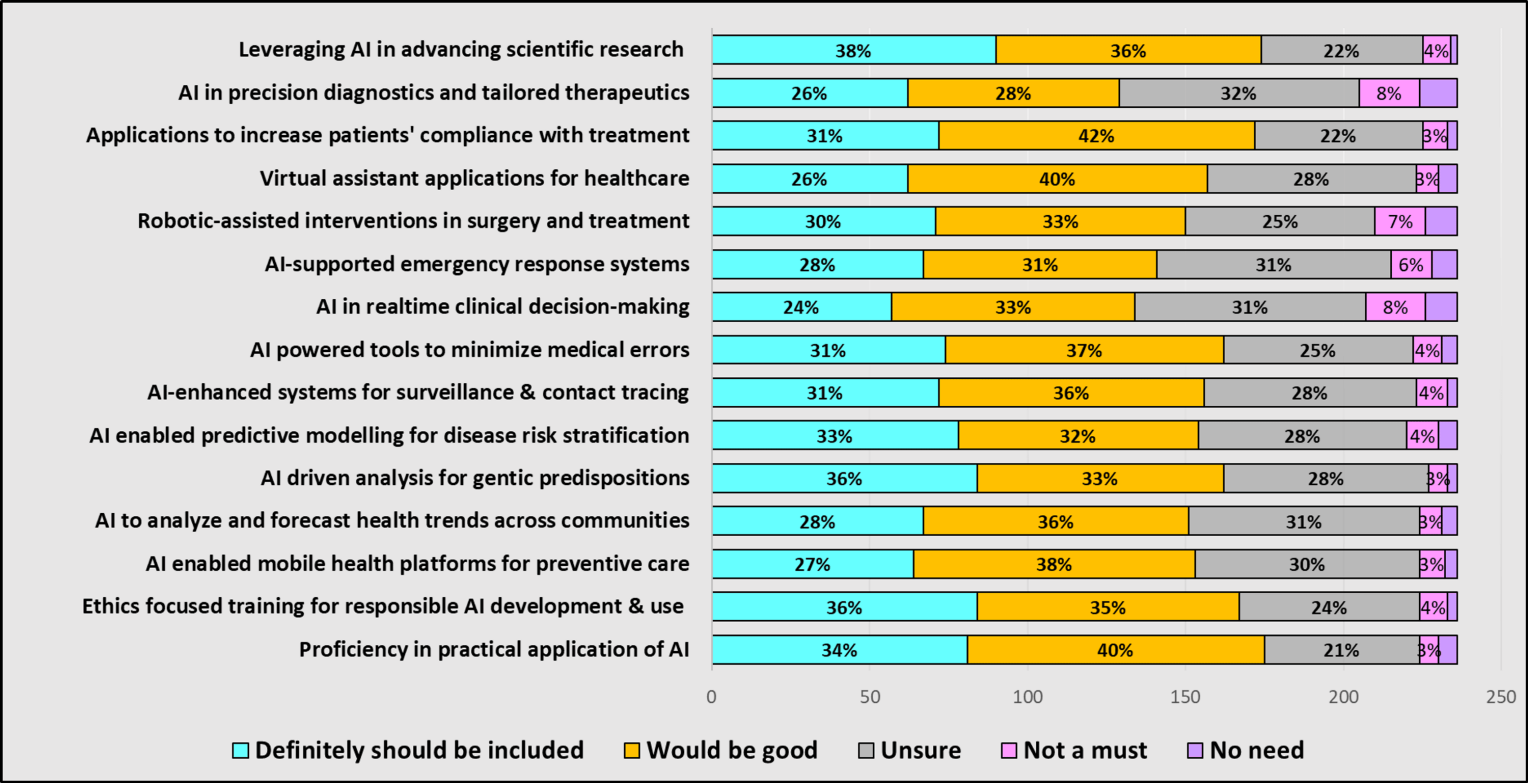
Opinions on suggested topics on AI to be included in the medical curriculum

Figure 3 presents respondents’ opinions on AI-related topics to be included in the medical curriculum. Overall, the majority favored inclusion, with most topics receiving over 60% combined endorsement for “Definitely should be included” or “Would be good.” Topics such as proficiency in practical application of AI, ethics-focused training, AI in scientific research, and AI-driven genetic predisposition analysis were among the most strongly supported. Even complex or advanced applications such as predictive modeling, robotic-assisted surgery, and AI for real-time decision-making received high support. Very few respondents chose “Not a must” or “No need” for any topic, indicating a strong overall consensus for integrating AI training across various clinical and research domains within the medical curriculum.

**Figure 4:**
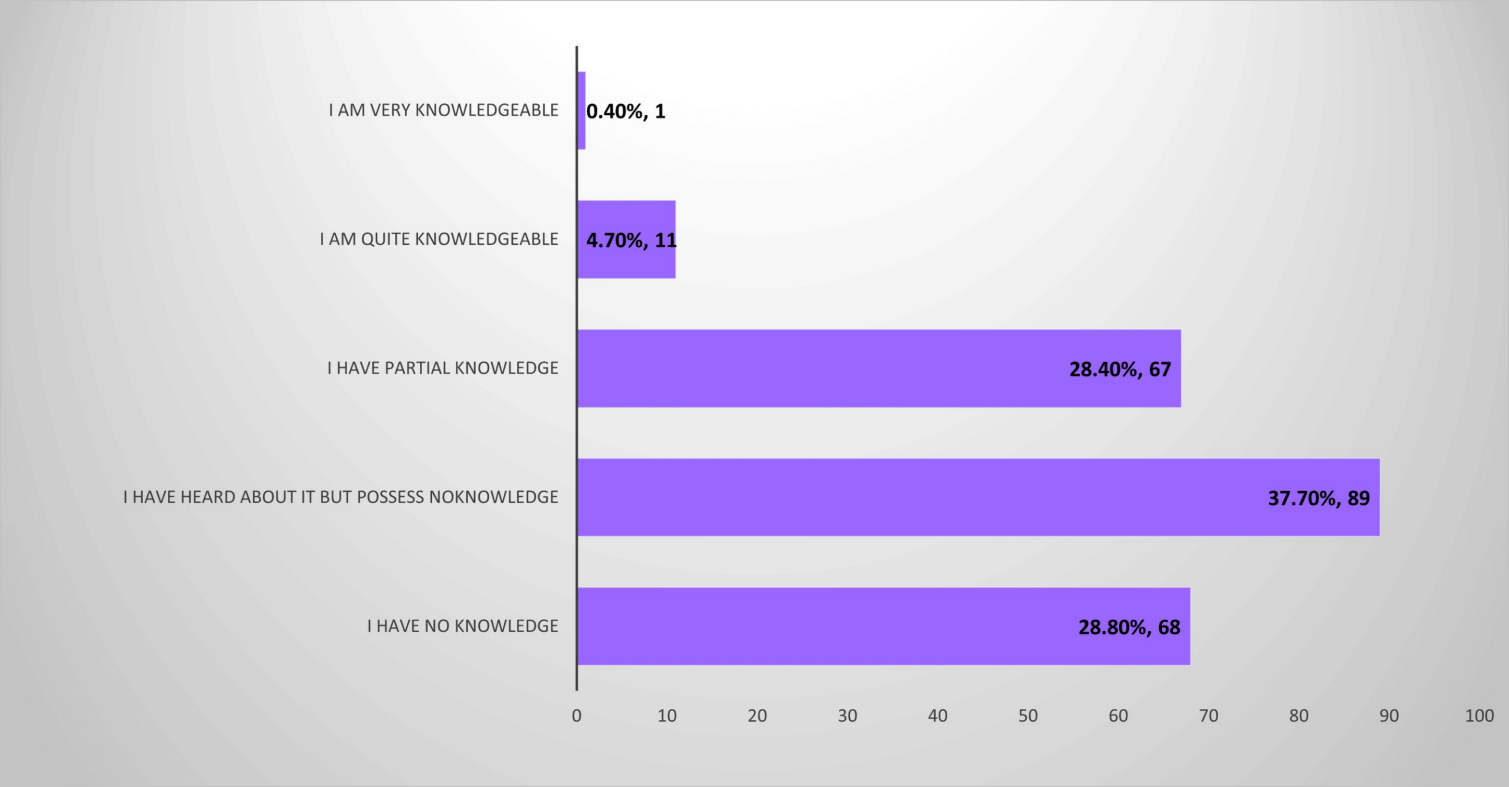
Self-reported knowledge levels of AI

Figure 4 presents respondents’ self-assessment of their prior knowledge related to AI. A large majority reported limited familiarity, with 89 (37.70%) stating they had heard about AI but possessed no real knowledge, and 68 (28.80%) admitting to having no knowledge at all. Another 67 (28.40%) described themselves as having only partial knowledge. Only a small fraction considered themselves quite knowledgeable (11, 4.70%), and just one (0.40%) claimed to be very knowledgeable. These findings highlight a substantial knowledge gap in AI among participants, underscoring the need for formal education and training in this area within the medical community.

**Figure 5:**
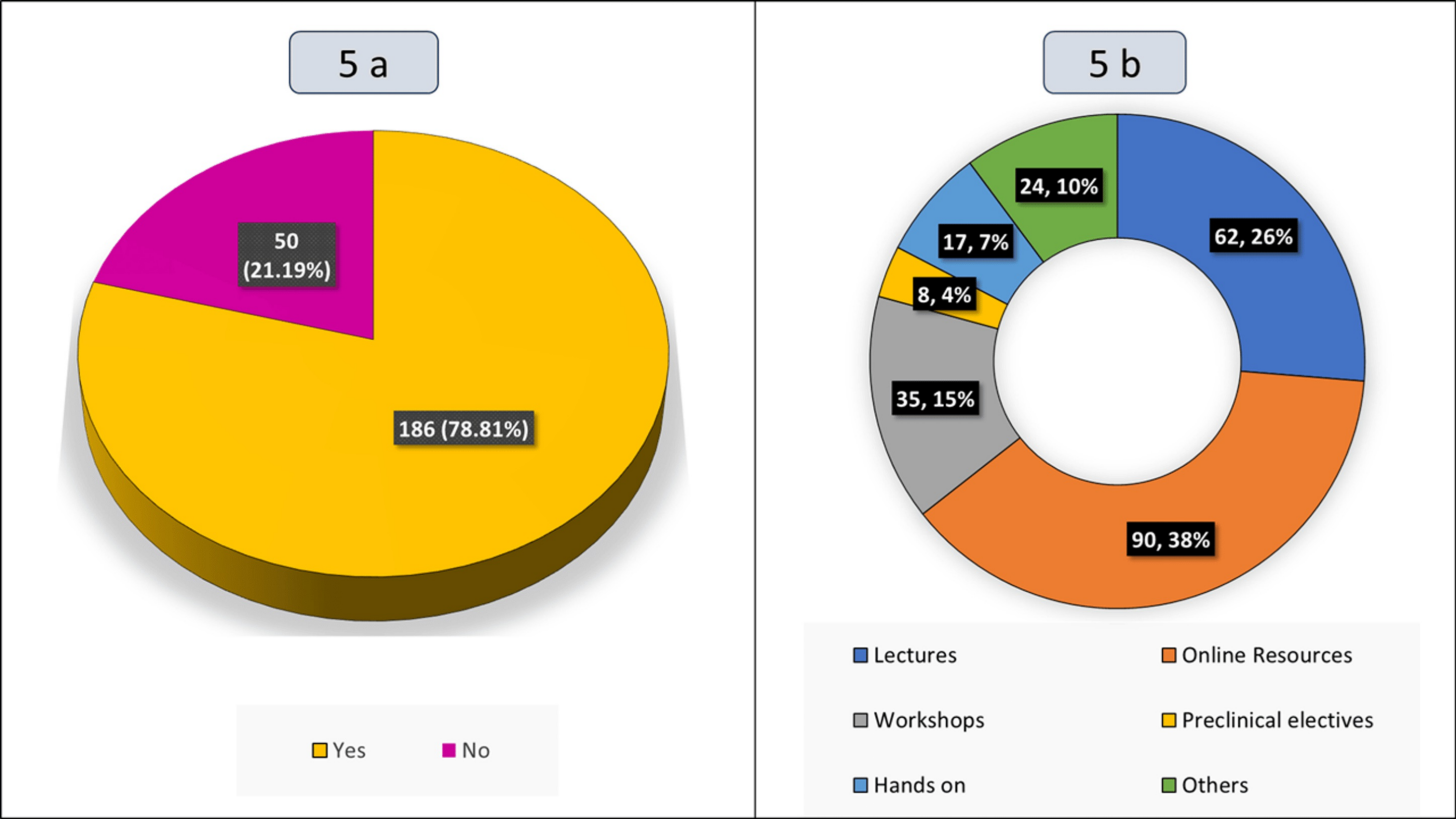
Response for training in AI and the format for learning

Figure 5a reveals that a substantial majority, 186 (78.81%), of respondents expressed willingness to receive training in AI during their medical education, indicating high interest and perceived relevance of AI in the medical field.

Figure 5b presents preferences for learning formats. The most preferred format was online resources (90, 38%), followed by lectures (62, 26%) and workshops (35, 15%). A smaller proportion favored hands-on training (17, 7%), preclinical electives (8, 4%), and other methods (24, 10%). These findings suggest a strong inclination toward flexible and self-paced digital learning, with moderate interest in interactive and structured formats.

**Figure 6:**
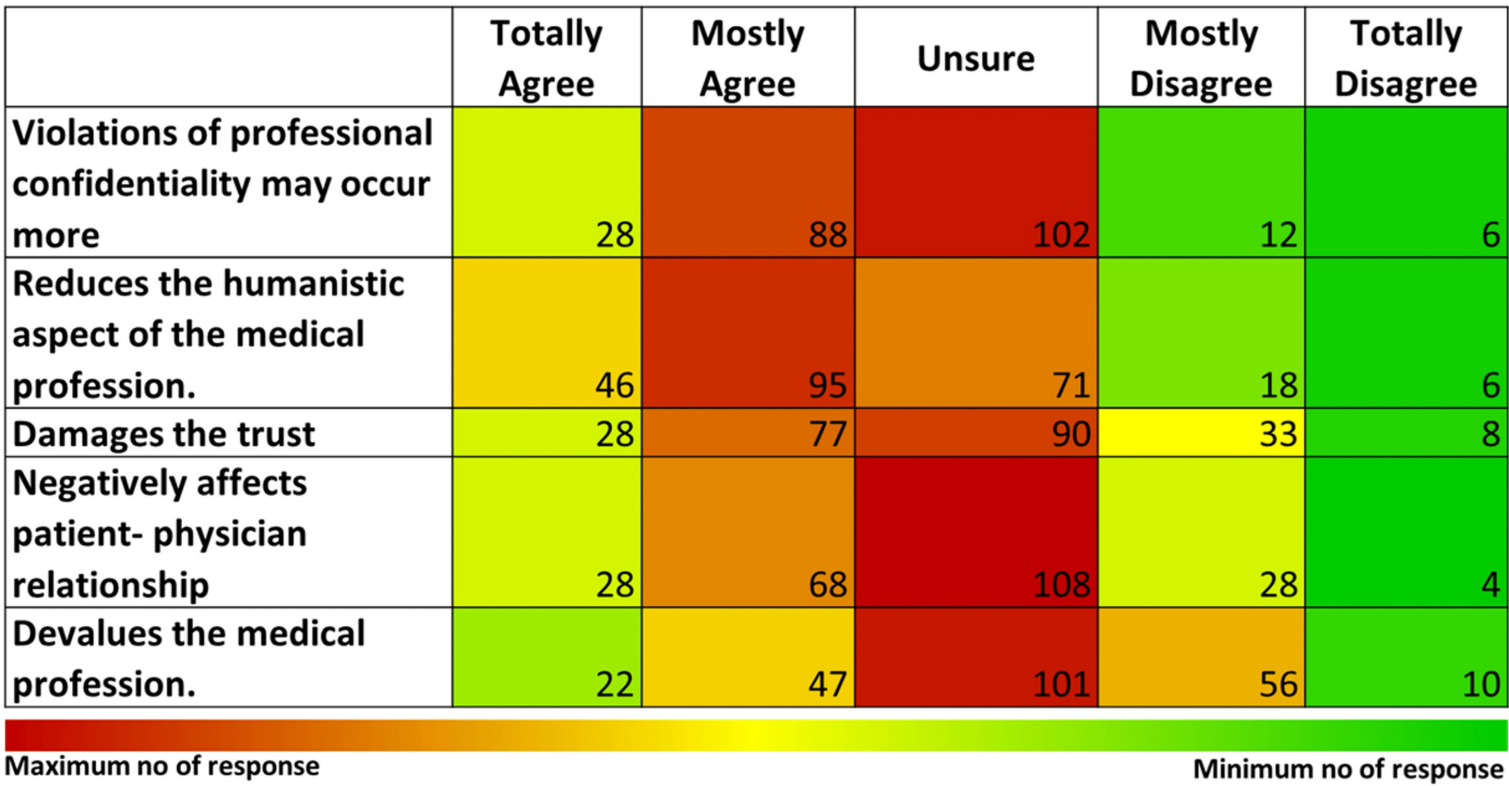
Heatmap showing opinions on ethical considerations of including AI in medicine

Figure 6 presents a heatmap illustrating participants’ opinions on ethical concerns related to the inclusion of AI in medicine. The most prominent concern was that AI might negatively affect the patient-physician relationship, with 108 respondents unsure and 96 agreeing to some extent. A significant number also believed that AI reduces the humanistic aspect (141 agreed) and damages trust (105 agreed). Concerns about violations of professional confidentiality and devaluation of the medical profession were present but relatively less pronounced. Across all statements, a considerable proportion of respondents were either unsure or expressed partial agreement, highlighting ambivalence and ethical concerns among future healthcare professionals regarding the integration of AI in medical practice.

## Discussion

This cross-sectional study offers a contemporary snapshot of Indian medical students’ perceptions, knowledge, and perceived training needs regarding AI in medicine and medical education. These findings mirror, reinforce, and extend global trends identified in earlier research from both high-income and developing countries [8-10].

Perceptions and attitudes toward AI

The majority of respondents expressed a generally favorable attitude toward the promise of AI to augment healthcare delivery, enhance clinical decision-making, and improve both patient safety and informational equity. This widespread optimism aligns with recent curriculum studies and expert consensus reports, which emphasize the unique capacity of AI to facilitate access to knowledge, support precision, reduce errors, and bridge geographical and resource divides within clinical systems. Notably, most Indian students did not perceive AI as a direct threat to the prestige or core values of the medical profession, a finding consistent with recent studies in Turkey, Germany, and Canada, where students viewed AI as assistive rather than adversarial [8-10].

However, substantial proportions of students voiced ethical concerns and apprehensions about the possible erosion of empathy, the human relationship in clinical care, and trust, echoing ambivalence seen globally. This duality (i.e., optimism for technological advancement tempered by a desire to preserve patient-centered care and ethical standards) is now a major theme in the international discourse on medical AI curricula.

Knowledge and training gaps

Despite pronounced enthusiasm, students in this study reported low self-rated knowledge and considerable gaps in formal training, paralleling trends observed in cross-national needs assessments and systematic reviews. The vast majority indicated only superficial familiarity with AI, with less than 15% reporting any prior formal exposure or structured education. These findings suggest that prior AI training was associated with more positive perceptions. This critical knowledge gap is widely documented and is one of the foremost barriers to effective AI integration in medical training and practice [9,11-13].

Importantly, students who had participated in some form of AI training expressed greater confidence, perceived AI more as an augmenting rather than replacing force, felt more optimistic about AI’s ability to support their future professional role, and demonstrated greater readiness for incorporating AI into the medical curriculum. These findings mirror international evidence: training is consistently associated with more nuanced, positive perceptions and a decreased fear of job displacement [14-16].

Demand for curricular reform

There was overwhelming consensus among participants in favor of integrating structured AI education, including fundamentals, practical skills, ethics, and advanced clinical applications, into the undergraduate curriculum. Calls for inclusion of AI literacy go beyond technical skills, encompassing ethical, legal, and clinical dimensions, and echo curricular frameworks emerging in the UK, Canada, and elsewhere. Globally, consensus statements and Delphi panel studies now list ethics, law, clinical application, and collaborative skills as the pillars of AI competency for future physicians.

Our participants’ strong support for integrated, contextually relevant AI content, including hands-on learning, research applications, and ethical decision-making, underscores the need for locally adapted but globally informed curricular frameworks. Their preference for online and flexible learning formats aligns with the growing global adoption of hybrid, self-paced digital programs [17-20].

Ethical and humanistic concerns

Consistent with previous research, students’ uncertainties and concerns clustered around potential impacts on professional identity, patient relationships, confidentiality, and the risk of dehumanization of care. Many remained ambivalent or unsure, reflecting both a need for more rigorous education and the ongoing negotiation of professional identity in an AI-augmented world.

Curricular reforms should therefore not only impart technical proficiency but also foster critical thinking, ethical literacy, and noncognitive professional competencies least likely to be replaced by AI, such as empathy, communication, and patient advocacy.

Limitations

This study has several limitations. First, it was conducted at a single tertiary care medical college using convenience sampling, which may limit the external validity of the findings. Therefore, the results should be interpreted cautiously and may not fully represent all Indian undergraduate medical students or institutions with differing educational contexts.

Second, although the response rate was high, a formal nonresponse analysis was not performed, and the characteristics of students who did not participate remain unknown. Third, the study relied on self-reported perceptions and knowledge, which may be subject to recall and social desirability bias.

Additionally, the study did not assess participants’ baseline digital literacy or differentiate between formal and informal sources of prior AI exposure, both of which could have influenced perceptions toward AI. Finally, while Likert-scale responses were treated primarily as ordinal data, parametric tests were applied to composite scores after assessment of approximate normality. Nevertheless, the use of parametric methods on ordinal-scale data carries inherent statistical assumptions that should be interpreted with caution.

Furthermore, the cross-sectional nature of the study represents an inherent limitation, as data were collected at a single time point, precluding any inference of causality between training exposure and participants’ perceptions. Future multicenter studies with more diverse participant populations, validated psychometric tools, and longitudinal designs are recommended to improve generalizability and better understand evolving perceptions regarding AI in medical education.

## Conclusions

This study confirms a dire need to bridge the substantial knowledge and training gap in AI among medical students, who demonstrate strong enthusiasm for AI integration despite limited formal exposure. Students with training exhibited more positive perceptions, viewing AI as an augmentative tool that enhances clinical decision-making, efficiency, and patient care rather than a threat to employment, while those without training were significantly more likely to fear job displacement. The overwhelming support for comprehensive curricular inclusion (over 60% endorsement for practical applications, ethics, and research uses) and preference for flexible online formats underscores students’ readiness and the necessity for structured, competency-based AI education that addresses both technical skills and ethical concerns, such as data privacy and the erosion of patient-physician relationships.

Educational reform should prioritize the spiral integration of AI literacy across the MBBS curriculum, beginning with foundational concepts and advancing toward practical clinical applications. This progression must be complemented by a sustained emphasis on humanistic competencies that remain beyond the scope of AI. Adopting blended learning approaches tailored to resource-constrained settings, such as Government Medical College, Nagpur, along with structured faculty development initiatives, will be essential. Such a balanced and context-sensitive strategy can equip future physicians to responsibly harness AI for precision medicine and equitable healthcare delivery while preserving the compassionate essence of medical practice.

## Appendices

Appendix A: Study questionnaire

Informed Consent

Please share a little of your time to answer this survey. This survey should take no more than 15 minutes to complete. There are no right or wrong answers. Your participation is voluntary, and you may withdraw from answering this survey at any time. Please read the questions and answer honestly.

I have read the foregoing information. By participating in this survey, I indicate that I am voluntarily participating in this study. I have given my consent to participate in this survey.

I agree with the statement above and participate in this survey.

Yes

No

Section A: Sociodemographic Details

1. Age (in completed years): ______

2. Gender: Male / Female / Prefer not to say

3. Current academic phase: First professional / Second professional / Third professional part I / Third professional part II / Intern

Section B: Prior Exposure and Knowledge of AI

1. Have you received any formal or informal training in AI? Yes / No

2. How would you rate your current knowledge of AI? No knowledge / Heard only / Partial / Quite knowledgeable / Very knowledgeable

Section C: Perceptions Regarding AI in Medicine

Response options:

0 = Strongly disagree

1 = Disagree

2 = Unsure

3 = Agree

4 = Strongly agree

AI can help patients make informed health-related choices.

AI erodes trust in the medical profession.

AI can strengthen health literacy among patients.

AI heightens vulnerability to patient data breaches.

AI erodes human connection in clinical care.

AI improves precision and reduces medical errors.

AI devalues the medical profession.

AI can help bridge geographical gaps in healthcare delivery.

AI undermines the compassionate dimension of care.

AI can improve trust in the healthcare system.

AI enhances doctors’ access to information.

AI can support clinical decision-making.

AI reduces the need for physicians and threatens employment opportunities.

AI cannot replace physicians but can support them.

AI applications can help me become a better doctor.

AI-powered tools can improve efficiency in maintaining patient records.

Section D: Ethical Concerns Related to AI

Response options:

Totally agree

Mostly agree

Unsure

Mostly disagree

Totally disagree

AI may damage trust between patients and doctors.

AI may devalue the medical profession.

AI may increase violations of professional confidentiality.

AI may negatively affect the patient-physician relationship.

AI may reduce the humanistic aspect of medicine.

Section E: Training Needs

Would you like to receive formal AI training during medical education?

Yes

No

Section F: Preferred Learning Format

Preferred mode for AI learning:

Online resources

Lectures

Workshops

Hands-on training

Preclinical electives

Other: ___________

Section G: AI Topics Suggested for Curriculum Inclusion

Response options:

No need

Not necessary

Unsure

Would be good to include

Definitely should be included

AI in scientific research

AI in precision diagnostics and tailored therapeutics

AI applications for improving treatment compliance

Virtual assistant applications in healthcare

Robotic-assisted surgery and interventions

AI-supported emergency response systems

AI in real-time clinical decision-making

AI-powered tools to reduce medical errors

AI-enhanced disease surveillance and contact tracing

Predictive modelling for disease risk stratification

AI-driven analysis of genetic predisposition

AI for forecasting community health trends

AI-enabled mobile health platforms for preventive care

Ethics-focused AI training

Practical applications of AI in medicine

Section E: Training Needs

Would you like to receive formal training in AI during medical education? Yes / No

Section F: Preferred Mode of Learning AI

 Preferred format: Online resources / Lectures / Workshops / Hands-on training / Preclinical electives / Other

Section G: AI Topics to be Included in Curriculum

AI in advancing scientific research

AI in precision diagnostics and tailored therapeutics

Applications of AI to increase patient compliance with treatment

Virtual assistant applications for healthcare

Robotic-assisted interventions in surgery and treatment

AI-supported emergency response systems

AI in real-time clinical decision-making

AI-powered tools to minimize medical errors

AI-enhanced systems for surveillance and contact tracing

AI-enabled predictive modelling for disease risk stratification

AI-driven analysis for genetic predispositions

AI to analyze and forecast health trends across communities

AI-enabled mobile health platforms for preventive care

Ethics-focused training for responsible AI development and use

Proficiency in the practical application of AI
